# The polymorphism rs3024505 proximal to IL-10 is associated with risk of ulcerative colitis and Crohns disease in a Danish case-control study

**DOI:** 10.1186/1471-2350-11-82

**Published:** 2010-05-28

**Authors:** Vibeke Andersen, Anja Ernst, Jane Christensen, Mette Østergaard, Bent A Jacobsen, Anne Tjønneland, Henrik B Krarup, Ulla Vogel

**Affiliations:** 1 Medical Department, Viborg Regional Hospital, DK-8800 Viborg, Denmark; 2 Institute of Cancer Epidemiology, Danish Cancer Society, DK-2100 Copenhagen, Denmark; 3 Department of Clinical Biochemistry, Aarhus University Hospital, DK-9100 Aalborg, Denmark; 4 Department of Medical Gastroenterology, Aarhus University Hospital, DK-9100 Aalborg, Denmark; 5 National Food Institute, Technical University of Denmark, DK-2860 Søborg, Denmark; 6 Institute for Science, Systems and Models, University of Roskilde, DK-4000 Roskilde, Denmark; 7 National Research Centre for the Working Environment, DK-2100 Copenhagen, Denmark; 8 Department of Clinical Biochemistry, Viborg Regional Hospital, DK-8800 Viborg, Denmark

## Abstract

**Background:**

Crohns disease (CD) and ulcerative colitis (UC) are characterized by a dysregulated inflammatory response to normal constituents of the intestinal flora in the genetically predisposed host. Heme oxygenase-1 (*HO-1/HMOX1*) is a powerful anti-inflammatory and anti-oxidant enzyme, whereas the pro-inflammatory interleukin 1β (*IL-1β/IL1B*) and anti-inflammatory interleukin 10 (*IL-10/IL10*) are key modulators for the initiation and maintenance of inflammation. We investigated whether single nucleotide polymorphisms (SNPs) in the *IL-1β*, *IL-10*, and *HO-1 *genes, together with smoking, were associated with risk of CD and UC.

**Methods:**

Allele frequencies of the *IL-1β *T-31C (rs1143627), and *IL-10 *rs3024505, G-1082A (rs1800896), C-819T (rs1800871), and C-592A (rs1800872) and *HO-1 *A-413T (rs2071746) SNPs were assessed using a case-control design in a Danish cohort of 336 CD and 498 UC patients and 779 healthy controls. Odds ratio (OR) and 95% confidence interval (95% CI) were estimated by logistic regression models.

**Results:**

Carriers of rs3024505, a marker polymorphism flanking the *IL-10 *gene, were at increased risk of CD (OR = 1.40, 95% CI: 1.06-1.85, P = 0.02) and UC (OR = 1.43, 95% CI: 1.12-1.82, P = 0.004) and, furthermore, with risk of a diagnosis of CD and UC at young age (OR = 1.47, 95% CI: 1.10-1.96) and OR = 1.35, 95% CI: 1.04-1.76), respectively). No association was found between the *IL-1β, IL-10 *G-1082A, C-819T, C-592A, and *HO-1 *gene polymorphisms and CD or UC. No consistent interactions between smoking status and CD or UC genotypes were demonstrated.

**Conclusions:**

The rs3024505 marker polymorphism flanking the *IL-10 *gene was significantly associated with risk of UC and CD, whereas no association was found between *IL-1β *or *HO-1 *gene polymorphisms and risk of CD and UC in this Danish study, suggesting that *IL-10*, but not *IL-1β *or *HO-1, *has a role in IBD etiology in this population.

## Background

The chronic inflammatory bowel diseases (IBD), ulcerative colitis (UC) and Crohn's disease (CD), are complex diseases caused by an interplay between genetic and environmental factors [1].

The recent years have brought much progress regarding the genetics in IBD and the number of confirmed IBD associated loci and genes have risen dramatically [2-7]. Yet, still, only part of the genetic contribution to disease risk may be explained by the identified genes [8,9]. Northern European populations, including the Danish, generally have low frequencies of the CD risk-associated variants of *CARD15 *[3,10], and it is therefore of interest to search for more genetic determinants in these populations. Less progress has been achieved in the identification of environmental risk factors and gene-environmental interactions. Differences in environmental exposures and genetic heterogeneity between ethnic groups may have complicated the search for genetic and gene-environmental determinants.

The emerging picture of IBD pathogenesis is focused on the sequential occurrence of pivotal events leading to the initiation and subsequent perpetuation of inflammation [11,12]. First, the initial interaction between luminal constituents and intestinal epithelial cells leads to activation of the innate immune system [11]. The recognition of highly conserved pathogen structures such as lipopolysaccharide (LPS), the main constituent of Gram-negative bacteria, by Toll-like receptors and other pattern recognition receptors on the epithelial and other immunologically active cells in the intestine, initiates the release of various cytokines and enzymes, including interleukins (IL) and heme oxygenase-1 [13,14]. Second, the inflammation will eventually become chronic due to defective regulation of the immune response. Therefore, polymorphisms in genes encoding cytokines and other molecules involved in the innate immune system, may affect the course of the inflammatory cascade and thereby the risk of developing IBD.

Activation of the pro-inflammatory IL-1β leads to production of prostaglandin E_2 _(PGE_2_) and nitric oxide (NO) via the induction of cyclo-oxygenase 2 (COX-2) and inducible nitric oxide synthase (iNOS) among others [15]. *IL-1β *knock-out mice have no spontaneous abnormalities, however, on challenge with LPS, a less pronounced acute phase response is observed, suggesting that IL-1β is required for an adequate immune response [15]. In both CD and UC patients, high levels of IL-1β are found in the intestinal mucosa [16] and stimulation by IL-1β leads to a more pronounced inflammatory response in CD immune cells compared to cells from healthy controls [17]. The variant alleles of two *IL-1β *promoter polymorphisms, *IL-1β *T-31C and *IL-1β *C-511T, have been found to be in almost complete linkage disequilibrium [18], and the haplotypes encompassing the *IL-1β *T-31C variant conferred higher transcription of *IL-1β *compared to the wild type haplotype [18]. The role of *IL-1β *polymorphisms in IBD has been explored in several studies [19-24]. These studies did not find any association with *IL-1β*, however, the studies were rather small.

IL-10 is an anti-inflammatory cytokine, which leads to dampening of the activated immune system. *IL-10 *knock-out mice develop colitis if they are not kept in germ-free environment [25], and the administration of IL-10 ameliorates the inflammation in animal and *in vitro *models [26]. In patients, an impaired IL-10 production has been fund in severe cases of CD and UC [27,28]. Recently, a strong association between the marker rs3024505 immediately downstream of the IL-10 gene and adult UC was found in a genome-wide association study [29]. This study also found a modest association between this marker and CD risk [29]. However, no association was found for rs3024493, a linked polymorphism located in an intron in the *IL-10 *gene, in a case-control study of paediatric onset CD [30]. The *IL-10 *promoter is polymorphic and genetic variation may account for different levels of cytokine production [31]. The *IL-10 *promotor polymorphisms G-1082A, C-819T, and C-592A have been most extensively studied. They are in tight linkage disequilibrium [32] and the haplotype encompassing these three polymorphisms is associated with low IL-10 protein production in lymphocytes *in vitro *[32] and low of levels of circulating IL-10 protein in Kenyan children [33] probably because the A allele of the *IL-10 *promoter polymorphism C-592A leads to the formation of a binding site for the ETS family of transcription factors [34]. Studies on the *IL-10 *promoter polymorphisms and IBD susceptibility have been inconsistent [22,29-31,35-40].

Heme oxygenase-1 (HO-1) is involved in the degradation of heme, thereby reducing oxidative stress and protecting against acute and chronic inflammation [41]. Animal models of IBD have confirmed the anti-inflammatory effect of HO-1 [42]. Hence, blockade of HO-1 activity results in exacerbation of experimental colitis whereas increased HO-1 pathway activity ameliorates experimental murine colitis [43-45]. Carbon monoxide (CO) is one of the main metabolite of the HO-1 pathway and CO administration has been shown to ameliorate chronic colitis in IL-10 deficient mice [42,46]. In UC patients, *HO-1 *expression and protein levels have been reported to be increased in inflamed colon compared to normal mucosa from patients with UC [47]. Studies using luciferase reporter assays of a functional promoter polymorphism, *HO-1 *A-413T, indicated that the A allele promoter had significantly higher activity than the T allele promoter [48]. The AA genotype of this polymorphism has been associated with a reduced incidence of ischemic heart disease [48]. Another promoter polymorphism, the *HO-1 *(GT)N dinucleotide repeat polymorphism, was not associated with risk of inflammatory bowel disease [49]. Interestingly, smoking affects the risk of IBD differentially, increasing the risk of CD and reducing the risk of UC [50]. The mechanisms by which smoking affects risk of IBD is not clear and as tobacco smoke contains more than 3000 chemicals, several different mechanisms may be involved. Nicotine has been reported to modulate the immune balance in a Th1-dominant direction [51] in accordance with the beneficial effect of smoking on UC. Moreover, smoking increases the production of certain pro-inflammatory cytokines, but, on the other hand, smoking is a source of carbon monoxide (CO). Thus, another possible mechanisms may involve interactions between smoking and IL-1β, IL-10 and HO-1 activity in relation to intestinal inflammation [46].

In this study we wanted to assess the role of polymorphisms in *IL-1β, IL-10*, and *HO-1 *together with smoking in relation to risk of developing IBD in a Danish case-control study of 336 CD, 498 UC and 779 healthy controls, respectively.

## Methods

### Patients and controls

Patients with CD (n = 373) or UC (n = 541), and healthy controls (n = 796) were included. All information was available for 336 CD cases, 498 UC cases and 779 healthy controls. Diagnosis of CD or UC was based on clinical, radiological, endoscopic and histological examinations [52]. Infectious and other cases of inflammatory bowel diseases were excluded. IBD diagnoses were classified on basis of disease localization according to the Montreal classification; L1, L2, and L3 signifies ileal, colonic, and ileocolonic localisation in CD and E1, E2, and E3 signifies the extent of colon involvement in UC; proctitis (inflammation limited to rectum), left side (inflammation distal to the left flexure), and extensive colon (inflammation proximal to the left flexure) [53]. Patients were recruited from Viborg, Aalborg and Herning Regional Hospitals from January 2004 to March 2005. Healthy blood donors were recruited from Viborg Hospital. Subjects under the age of 18 and with ethnicity other than Caucasian were excluded from the study. Phenotypic data on age at diagnosis of disease, localisation, family disposition for IBD, and medical and surgical treatment of disease was collected. Information on smoking habits at the time of diagnosis (patients) and smoking habits at study entry (donors) was collected.

### Genotyping

DNA was extracted from EDTA-stabilized peripheral blood samples from all patients and healthy controls by using either a PureGene (Gentra Systems, Minneapolis, MN, USA) or Wizard Genomic (Promega, Madison, WI, USA) DNA purification kit, according to the manufacturers' recommendations.

SNPs were chosen from literature studies. *IL-1β *T-31C (rs1143627), and *IL-10 *C-592T (rs1800872) were genotyped as previously described [54] Rs3024505, *IL-10 *G-1082A (rs1800896), C-819T (rs1800871) [29] were genotyped using the pre-developed allelic discrimination assays C_15983681 20, C_1747360_10 and C_1747362_10 (Applied Biosystems). The genotyping reaction was performed in a final volume of 6 μl consisting of 3 μl Universal PCR Master Mix, 0.075 μl 40 × assay-on-demand mix, 1.925 μl H_2_O, and 1 μl genomic DNA. *HO-1 *A-413T (rs2071746 was determined using the pre-developed allelic discrimination assay C_15869717_10 (Applied Biosystems). Genotyping was performed by TaqMan real-time PCR on an ABI7900HT (Applied Biosystems), using Allelic Discrimination. Twenty ng of DNA was genotyped in 5 μl containing 1 × Mastermix (Applied Biosystems, Nærum, Denmark), 100 nM probes, and 900 nM primers or as recommended by the manufacturer for predesigned assays.. Controls of known genotypes were included in each run, and repeated genotyping of a random 10% subset yielded 100% identical genotypes. Laboratory personnel were blinded to the case/control status of the study group [55].

### Statistical analyses

We used logistic regression to analyse the relationship between the six polymorphisms and disease. The statistical analyses included only subjects where all information was available. Age was entered linear in the model after checking for linearity using a linear spline [56]. Subgroup analyses were done for the polymorphisms in relation to location of the disease (CD: L1, L2, L3, UC: E1, E2, E3), and age at diagnosis (above or not above 40 years of age) for the cases. The GENMOD procedure in SAS release 9.1 (SAS Institute, Inc., Cary, North Carolina, USA) was used for the statistical analyses.

### Power analyses

We used Genetic Power Calculator for Case - control for discrete traits [57] for power analyses. This study has more than 80% power to detect a dominant effect with an OR of 1.5 in either CD or in UC or 1.4 if CD and UC were combined.

### Ethical Considerations

All subjects received written and oral information and gave written informed consent. The study was conducted in accordance with the Declaration of Helsinki and approved by the local Scientific Ethical Committees at Viborg and Aalborg County (VN 2003/5).

## Results

Characteristics of the Danish IBD patients and controls are shown in Table 1. A total of 834 Danish patients and 779 controls were included. 51% of the CD patients were current smokers at the time of diagnosis, whereas only 17% of the UC patients were current smokers. The genotype distributions among the controls did not deviate from Hardy-Weinberg equilibrium. The variant allele frequencies for *IL-1β *T-31C, *IL-10 *rs3024505, G-1082A, C-819T, C-592A and *HO-1 *A-413T were 0.35, 0.18, 0.45, 0.21, 0.21, and 0.42, respectively, in the control group.

**Table 1:** The basic descriptions of the Danish study subjects^1^.

	Crohns Disease	Ulcerative Colitis	Controls
	(n = 336)	(n = 498)	(n = 779)
**Gender**: n (%)			
male	131 (39)	241 (48)	397 (51)
female	205 (61)	257 (52)	382 (49)
**Age**:			
Median (5%-95%)	43 (23-77)	49 (24-76)	43 (23-60)
**Age at diagnosis**:			
Median (5%-95%)	30 (15-65)	35 (17-68)	
**Smoking habits**: n(%)			
Smokers	170 (51)	85 (17)	204 (26)
Never smokers	120 (36)	231 (46)	391 (50)
Former smokers	46 (14)	182 (37)	184 (24)
**Location UC**^**2**^:			
Proctitis (E1)		211 (42)	
Left side (E2)		183 (37)	
Extensive (E3)		94 (19)	
Data not available		10 (2)	
**Location CD**^**2**^: n (%)			
Colonic (L2)	75 (22)		
Ileal (L1)	155 (46)		
Ileocolonic (L3)	93 (28)		
Data not available	13 (4)		
**Medication**: n (%)			
Advanced^3^	142 (42)	104 (21)	
No advanced medication^4^	189 (56)	390 (78)	
Data not available	5 (1)	4 (1)	
**Operation**: n (%)			
Yes	153 (46)	15 (3)	
No	176 (52)	473 (95)	
Data not available	7 (2)	10 (2)	

^1^The analyses included subjects where all information was available,

^2^The location of CD and UC in accordance with the Montreal classification are shown.

^3^azathioprin, 6-mercaptopyrine, Tumor Necrosis Factor-inhibitors, or methrotrexate,

^4^5-aminosalicylic acid, prednisolone

### Associations between polymorphisms and disease

Carriers of the variant allele of rs3024505 flanking the *IL-10 *gene were at increased risk of both CD and UC. Homozygous variant allele carriers were at 2.48-fold (95% CI: 1.27-4.84) increased risk of CD and heterozygous carriers were at 1.31-fold (95% CI: 0.98-1.75) increased risk of CD after adjusting for age, gender and smoking status (Table 2). Homozygous variant allele carriers were at 2.31-fold (95% CI: 1.27-4.20) increased risk of UC and heterozygous carriers were at 1.34-fold (95% CI: 1.04-1.73) increased risk of UC after adjusting for age, gender and smoking status (Table 3). After correction for multiple testing the associations were borderline statistically significant. Minor allele frequencies of *IL-10 *gene polymorphisms in selected studies are shown in Table 4.

**Table 2:** Genotypes in Danish patients with Crohns Disease^1^.

	**N**_**case**_	**N**_**control**_	**OR (95%CI)**^**2**^		**OR (95%CI)**^**3**^		**OR (95%CI)**^**4**^		**P-value**^**5**^
**IL1 β C-31T (rs1143627)**									
TT	165	342	1.00	-	1.00	-	1.00	-	
CT	139	342	0.84	(0.64-0.10)	0.85	(0.65-1.12)	0.86	(0.65-1.14)	0.29
CC	32	95	0.70	(0.45-1.09)	0.71	(0.45-1.10)	0.69	(0.44-1.09)	0.11
CT and CC	171	437	0.81	(0.63-1.05)	0.82	(0.63-1.07)	0.82	(0.63-1.07)	0.15
**IL-10 C-592A (rs1800872)**									
CC	214	483	1.00	-	1.00	-	1.00	-	
AC	114	261	0.99	(0.75-1.29)	0.98	(0.75-1.30)	1.01	(0.76-1.34)	0.97
AA	8	35	0.52	(0.24-1.13)	0.53	(0.24-1.16)	0.54	(0.24-1.21)	0.13
AC and AA	122	296	0.93	(0.71-1.21)	0.93	(0.71-1.22)	0.95	(0.72-1.25)	0.72
**IL-10 C-819T (rs1800871)**									
CC	216	483	1.00	-	1.00	-	1.00	-	
CT	111	259	0.96	(0.73-1.26)	0.95	(0.72-1.25)	0.97	(0.73-1.29)	0.83
TT	9	37	0.54	(0.26-1.15)	0.55	(0.26-1.18)	0.56	(0.26-1.22)	0.14
CT and TT	120	296	0.91	(0.69-1.18)	0.90	(0.69-1.18)	0.92	(0.70-1.21)	0.55
**IL-10 G-1082A (rs1800896)**									
GG	109	238	1.00	-	1.00	-	1.00	-	
AG	171	374	1.00	(0.75-1.33)	0.99	(0.74-1.33)	0.97	(0.72-1.32)	0.87
AA	56	167	0.73	(0.50-1.07)	0.75	(0.51-1.10)	0.78	(0.53-1.16)	0.23
AG and AA	227	541	0.92	(0.70-1.21)	0.92	(0.69-1.21)	0.92	(0.69-1.22)	0.56
**(rs3024505)**									
CC	203	522	1.00	-	1.00	-	1.00	-	
CT	114	235	1.25	(0.95-1.64)	1.27	(0.96-1.68)	1.31	(0.98-1.75)	0.07
TT	19	22	2.22	(1.18-4.19)	2.60	(1.36-4.96)	2.48	(1.27-4.84)	0.01
CT and TT	133	779	1.33	(1.02-1.73)	1.37	(1.05-1.80)	1.40	(1.06-1.85)	0.02
**HO-1 A-413T (rs2071746)**									
AA	110	267	1.00	-	1.00	-	1.00	-	
AT	165	373	1.07	(0.81-1.43)	1.06	(0.79-1.42)	1.05	(0.78-1.42)	0.75
TT	61	139	1.07	(0.73-1.55)	1.11	(0.76-1.62)	1.04	(0.70-1.53)	0.86
AT and TT	226	512	1.07	(0.82-1.41)	1.08	(0.82-1.42)	1.05	(0.79-1.39)	0.75

^1^OR = Odds Ratio, 95%CI = 95% confidence interval,

^2 ^Crude,

^3 ^Adjusted for age and gender,

^4 ^Adjusted for age, gender and smoking status.

^5^P for the fully adjusted estimate

**Table 3:** Genotypes in Danish patients with ulcerative colitis^1^.

	**N**_**case**_	**N**_**control**_	**OR (95%CI)**^**2**^		**OR (95%CI)**^**3**^		**OR (95%CI)**^**4**^		**P-value**^**5**^
**IL1 β C-31T (rs1143627)**									
TT	204	342	1.00	-	1.00	-	1.00	-	
CT	238	342	1.17	(0.92-1.48)	1.15	(0.90-1.47)	1.15	(0.89-1.47)	0.28
CC	56	95	0.99	(0.68-1.43)	0.99	(0.68-1.46)	1.02	(0.69-1.49)	0.94
CT and CC	294	437	1.13	(0.90-1.42)	1.11	(0.88-1.41)	1.12	(0.88-1.42)	0.35
**IL-10 C-592A (rs1800872)**									
CC	328	483	1.00	-	1.00	-	1.00	-	
AC	149	261	0.84	(0.66-1.07)	0.83	(0.65-1.07)	0.83	(0.64-1.07)	0.14
AA	21	35	0.88	(0.51-1.55)	0.99	(0.56-1.76)	1.00	(0.56-1.77)	0.99
AC and AA	170	296	0.85	(0.67-1.07)	0.85	(0.67-1.08)	0.85	(0.66-1.08)	0.18
**IL-10 C-819T (rs1800871)**									
CC	325	483	1.00	-	1.00	-	1.00	-	
CT	151	259	0.87	(0.68-1.11)	0.86	(0.67-1.11)	0.85	(0.66-1.10)	0.22
TT	22	37	0.88	(0.51-1.53)	1.00	(0.57-1.74)	1.00	(0.57-1.75)	0.99
CT and TT	173	296	0.87	(0.69-1.10)	0.88	(0.69-1.12)	0.87	(0.68-1.11)	0.27
**IL-10 G-1082A (rs1800896)**									
GG	169	238	1.00	-	1.00	-	1.00	-	
AG	239	374	0.90	(0.70-1.16)	0.91	(0.70-1.19)	0.93	(0.72-1.22)	0.61
AA	90	167	0.76	(0.55-1.05)	0.78	(0.56-1.08)	0.76	(0.54-1.06)	0.10
AG and AA	329	541	0.86	(0.67-1.09)	0.87	(0.68-1.12)	0.88	(0.68-1.13)	0.31
**(rs3024505)**									
CC	297	522	1.00	-	1.00	-	1.00	-	
CT	172	235	1.29	(1.01-1.64)	1.35	(1.05-1.73)	1.34	(1.04-1.73)	0.02
TT	29	22	2.32	(1.31-4.11)	2.37	(1.31-4.29)	2.31	(1.27-4.20)	0.01
CT and TT	201	779	1.37	(1.09-1.74)	1.43	(1.13-1.83)	1.43	(1.12-1.82)	0.004
**HO-1 A-413T (rs2071746)**									
AA	162	267	1.00	-	1.00	-	1.00	-	
AT	251	373	1.11	(0.86-1.43)	1.10	(0.85-1.42)	1.11	(0.85-1.44)	0.45
TT	85	139	1.01	(0.72-1.41)	1.00	(0.71-1.41)	1.01	(0.71-1.42)	0.98
AT and TT	336	512	1.08	(0.85-1.37)	1.07	(0.84-1.37)	1.08	(0.84-1.38)	0.55

^1^OR = Odds Ratio, 95%CI = 95% confidence interval,

^2 ^Crude,

^3 ^Adjusted for age and gender,

^4 ^Adjusted for age, gender and smoking status.

^5^P for the fully adjusted estimate

**Table 4:** Odds ratios and 95% confidence intervals (OR (CI)) for associations between IL-10 gene polymorphisms and ulcerative colitis (UC) or Crohns disease (CD) in selected case-control studies^3^.

**N**_**cases/control**_	**Trs3024505C**^**1**^	**Grs3024493T**^**1**^	**C-819T**^**2 **^ (rs1800871)	**C-592A**^**2 **^ (rs1800872)	**G-1082A**^**2 **^ (rs1800896)	Crs2222202T	
**UC**							
1855/3091	1.46 (1.31-1.62)		Neg	Neg	neg		[29]
203/391			neg		1.66 (1.30-2.14)		[36]
**CD**							
1848/1804	1.17 (1.01-1.34)						[29]
270/336		neg	0.77 (0.58-1.00)			1.29 (1.01-1.64)	[30]
234-6/188-231				neg	neg		[35]

^1^rs3024505 and rs3024493 are in complete linkage disequilibrium [30]

^2^*IL-10 *G-1082A, C-819T, and C-592A are in linkage disequilibrium [32] C-819T, and C-592A are in complete linkage disequilibrium [34]

^3^Negative associations are indicated by "Neg"

No association was found between the *IL-1β*, *IL-10 *G-1082A, C-819T, C-592A,, and *HO-1 *polymorphisms and risk of CD or UC (Table 2 and 3). The three *IL-10 *promoter polymorphisms were found to be in almost complete linkage as previously described for Caucasians [32]. Therefore, no haplotype analyses were performed.

No significant difference in the genotype distribution between CD and UC was found (data not shown). When combining UC and CD data to increase the statistical power there were still no associations between the *IL-1β*, the three *IL-10 *promoter polymorphisms, and *HO-1 *gene polymorphisms and risk of IBD (results not shown).

Subgroup analyses showed that variant allele carriers of rs3024505 were at 1.47-fold (95% CI: 1.10-1.96) and 1.35-fold (95% CI: 1.04-1.76) higher risk of a diagnosis of CD and UC, respectively, before the age of 40 years than the homozygous wildtype carriers (results not shown). No associations between rs3024505 genotype and disease localisation, or between *IL-1β*, the three *IL-10 *promoter polymorphisms, and *HO-1 *polymorphisms and age at diagnosis or disease localisation were found.

### Gene-smoking interaction analyses

The effect of smoking habits at diagnosis on the genotype associations was investigated for CD and UC, respectively (Additional file 1: Interaction between the studied polymorphisms and smoking status in relation to risk of Crohns Disease and Additional file 2: Interaction between the studied polymorphisms and smoking status in relation to risk of ulcerative colitis). No consistent interactions between smoking status and any of the genotypes were found.

## Discussion

The present case-control study showed that the rs3024505 marker polymorphism flanking the *IL-10 *gene was significantly associated with risk of CD and UC, and, furthermore, with risk of a diagnosis of CD and UC at young age. None of the polymorphisms *IL-1β *T-31C, *IL-10 *G-1082A, C-819T, C-592A, or *HO-1 *A-413T were associated with risk of CD, UC, or UC and CD combined. No consistent interactions between smoking status and genotypes were found.

Our results replicate the findings by Franke et al. [29] (Table 4). In addition, we found that the association was carried by a stronger association in the younger age group. Franke et al found that the variant allele of rs3024505 was associated with increased risk of UC with OR of 1.46 (95% CI: 1.31-1.62) and with CD with OR of 1.17 (95% CI: 1.01-1.34). Furthermore, they found no association between the three *IL-10 *promoter polymorphisms and risk of UC (results regarding CD were not reported). Previous studies were unable to find association between IBD and the *IL-10 *promoter polymorphisms [22,31,35,39,40] whereas other studies have found associations between paediatric onset of CD and *IL-10 *C-819T wildtype allele [30], Crs2222202T variant allele [30] and between the *IL-10 *G-1082A variant allele and risk of UC [58] (Table 4).

The biological significance of rs3024505 in IBD remains unclear [29]. The polymorphism is is located in an intergenic region proximal to the 3'UTR end of the *IL-10 *gene. The region has a high potential for containing regulatory sequences, and may thus regulate *IL-10 *gene expression [29]. Furthermore, rs3024505 is in perfect linkage with other polymorphisms located within the *IL-10 *gene [29]. On the other hand, since no associations were found between risk of UC or CD and the *IL-10 *promoter polymorphisms with proven functional effects on the *IL-10 *gene expression, this may suggest either that the rs3024505 has a much stronger regulatory effect on IL-10 levels than the promoter polymorphisms or that the effect of the polymorphism on disease risk is unrelated to IL-10 expression.

IL-1β, IL-10 and HO-1 are key players in the homeostasis of the intestinal immune system. Due to their pro-inflammatory and anti-inflammatory effects they are of significance for the development and maintenance of chronic inflammation. IL-1β has pro-inflammatory effects, whereas IL-10 and HO-1 have anti-inflammatory effects. A substantial number of studies document the roles of the interleukins, including IL-1β and IL-10, and HO-1 in intestinal inflammation in various animal IBD models and in IBD patients [15,25,26,43-47]. Therefore, genetic variations in these genes may cause imbalance in intestinal homeostasis and thereby contribute to chronic inflammation. On this background, *IL-1β, IL-10 *and *HO-1 *are relevant candidates for IBD susceptibility genes.

Our results are in accordance with previous studies which were unable to find association between IBD and *IL-1β *T-31C [24], taqI [19,23] or C-511T [20,21]. The *HO-1 *A-413T polymorphism has not previously been studied in relation to IBD, whereas no association was found between IBD and *HO-1 *(GT)N [49]. However, all these studies were small, the largest studies included 500 participants, and thus with limited statistical power to exclude an association. The polymorphisms analysed in the present study, *IL-1β *T-31C, and *HO-1 *A-413T have been shown to have biological effect [18,48,59], and the SNPs have previously been associated to risk of various disease entities [48,60].

We found no consistent interactions between the studied polymorphisms and smoking in relation to risk of CD or UC. Although both smoking and nicotine administration lower the exaggerated IL-1β response in IBD patients [61,62], the present study does not indicate that smoking at the time of diagnosis influences IBD risk by pathways involving *IL-1β*, *IL-10 *or *HO-1*, since the polymorphisms had no effect among present smokers. Cigarette smoke has been reported to act differentially on inflammation in the small and large intestine, thus worsening small intestinal inflammation, but ameliorating colitis [63]. We were not able to perform subgroup analyses to target this question due to limited statistical power.

It is important to stress the strengths and limitations of the study. The present study included 1600 participants and power analyses showed that this study has more than 80% power to detect a dominant effect with an OR of 1.5 in relation to either CD or UC and or 1.4 if CD and UC were combined. Moreover, genetic determinants may be stronger among patients with extensive disease and ileal disease [64,65] and disease onset at low age. The effects of the polymorphisms might thus be below the detection level of our study.

## Conclusions

In conclusion, the rs3024505 marker polymorphism flanking the *IL-10 *gene was associated with risk of UC and CD in the present Danish case-cohort study, and, furthermore, with risk of a diagnosis of CD and UC at young age. None of the polymorphisms *IL-1β *T-31C, *IL-10 *G-1082A, C-819T, C-592A, or *HO-1 *A-413T were associated with risk of CD or UC. No consistent interactions between smoking status and genotypes were found. The study suggests that *IL-10*, but not *IL-1β *or *HO-1, *play a role in IBD etiology.

## Abbreviations

CD: Crohns disease; CI: confidence interval; CO: carbon monoxide; COX-2: cyclooxygenase 2; HO-1: heme oxygenase 1; IBD: inflammatory bowel disease; IL-1β: interleukin 1β; IL-10: interleukin 10; iNOS: inducible nitric oxide synthase; NO: nitric oxide; OR: odds ratio; RQ-PCR: real-time quantitative RT-PCR; SNP: single nucleotide polymorphism; UC: ulcerative colitis; PGE_2_: prostaglandin E2

## Competing interests

The authors declare that they have no competing interests.

## Authors' contributions

UV and AE carried out the genotyping. VA, HK, AE, MØ, BAJ established the cohort and/or participated in sample preparation and collection. JC and AT performed the statistical analyses. VA and UV conceived the genotyping study, and its design and coordination and wrote the manuscript. All authors read and approved the final manuscript.

## Pre-publication history

The pre-publication history for this paper can be accessed here:

http://www.biomedcentral.com/1471-2350/11/82/prepub

## Supplementary Material

Additional file 1 **Interaction between the studied polymorphisms and smoking status in relation to risk of Crohns Disease**. Table. Click here for file

Additional file 2 **Interaction between the studied polymorphisms and smoking status in relation to risk of ulcerative colitis**. Table. Click here for file
