# Supplementary material for: The polymorphism rs3024505 proximal to IL-10 is associated with risk of ulcerative colitis and Crohns disease in a Danish case-control study
Source: BMC Med Genet. 2010 May 28;11:82. doi: 10.1186/1471-2350-11-82 (PMC2891714; doi:10.1186/1471-2350-11-82)
Supplement: Additional file 1 — Interaction between the studied polymorphisms and smoking status in relation to risk of Crohns Disease. Table. [file 1471-2350-11-82-S1.PDF]

Additional file 1: Interaction between the studied polymorphisms and smoking status in relation to risk of Crohns Disease.

	Smoking						Smoking						
	Never		Past		Current		Never		Past		Current		
	N_{cases}	$N_{controls}$	N_{cases}	$N_{controls}$	N_{cases}	$N_{controls}$	OR (95%CI)[B]		OR (95%CI)[B]		OR (95%CI)[B]		p-value[A]
**IL1 β C-31T (rs1143627)**													
TT	19	89	60	166	86	87	1.00	-	0.54	(0.30-0.97)	2.58	(1.69-3.95)	0.30
CT and CC	27	95	60	225	84	117	0.74	(0.49-1.13)	0.71	(0.42-1.21)	1.93	(1.28-2.92)	
**IL-10 C-592A (rs1800872)**													
CC	27	108	82	242	105	133	1.00	-	0.64	(0.39-1.06)	2.19	(1.52-3.15)	0.29
AC and AA	19	76	38	149	65	71	0.73	(0.47-1.13)	0.69	(0.39-1.22)	2.57	(1.68-3.94)	
**IL-10 C-819T (rs1800871)**													
CC	27	106	84	244	105	133	1.00	-	0.64	(0.39-1.06)	2.17	(1.51-3.11)	0.24
CT and TT	19	78	36	147	65	71	0.69	(0.44-1.07)	0.66	(0.37-1.16)	2.51	(1.64-3.84)	
**IL-10 G-1082A (1800896)**													
GG	12	54	41	123	56	61	1.00	-	0.57	(0.28-1.19)	2.67	(1.60-4.46)	0.65
AG and AA	34	130	79	268	114	143	0.88	(0.57-1.37)	0.72	(0.43-1.22)	2.27	(1.46-3.51)	
**Rs3024505**													
CC	31	123	69	258	103	141			0.88	(0.54-1.43)	2.62	(1.80-3.81)	0.46
CT and TT	15	61	51	133	67	63	1.52	(0.99-2.32)	0.82	(0.44-1.55)	4.00	(2.57-6.22)	
**HO-1 A-413T (rs2071746)**													
AA	20	55	34	148	56	64	1.00	-	1.41	(0.74-2.68)	3.75	(2.22-6.33)	0.04
At and TT	26	129	86	243	114	140	1.56	(0.99-2.45)	0.81	(0.46-1.44)	3.38	(2.15-5.33)	

[A]P for interaction

[B] Adjusted for age and gender.
