# Supplementary material for: The polymorphism rs3024505 proximal to IL-10 is associated with risk of ulcerative colitis and Crohns disease in a Danish case-control study
Source: BMC Med Genet. 2010 May 28;11:82. doi: 10.1186/1471-2350-11-82 (PMC2891714; doi:10.1186/1471-2350-11-82)
Supplement: Additional file 2 — Interaction between the studied polymorphisms and smoking status in relation to risk of ulcerative colitis. Table. [file 1471-2350-11-82-S2.PDF]

Additional file 2: Interaction between the studied polymorphisms and smoking status in relation to risk of ulcerative colitis.

	**Smoking**						**Smoking**						
	Never		Past		Current		Never		Past		Current		
	N_{cases}	$N_{controls}$	N_{cases}	$N_{controls}$	N_{cases}	$N_{controls}$	OR (95%CI)[B]		OR (95%CI)[B]		OR (95%CI)[B]		[A]p-value
**IL1 β C-31T (rs1143627)**													
TT	64	89	106	166	34	87	1.00	-	0.85	(0.56-1.29)	0.55	(0.34-0.89)	0.05
CT and CC	118	95	125	225	51	117	0.87	(0.62-1.22)	1.47	(1.01-2.15)	0.59	(0.39-0.90)	
**IL-10 C-592A (rs1800872)**													
CC	120	108	158	242	50	133	1.00	-	1.28	(0.91-1.81)	0.51	(0.34-0.75)	0.20
AC and AA	62	76	73	149	35	71	0.76	(0.53-1.08)	0.96	(0.64-1.44)	0.67	(0.42-1.06)	
**IL-10 C-819T (rs1800871)**													
CC	120	106	156	244	49	133	1.00	-	1.34	(0.95-1.89)	0.51	(0.34-0.75)	0.14
CT and TT	62	78	75	147	36	71	0.81	(0.57-1.15)	0.95	(0.64-1.43)	0.71	(0.45-1.12)	
**IL-10 G-1082A (1800896)**													
GG	66	54	77	123	26	61	1.00	-	1.39	(0.86-2.23)	0.59	(0.34-1.03)	0.84
AG and AA	116	130	154	268	59	143	0.90	(0.63-1.29)	1.09	(0.74-1.62)	0.57	(0.37-0.88)	
**Rs3024505**													
CC	109	123	139	258	49	141	1.00	-	1.27	(0.90-1.80)	0.58	(0.39-0.85)	0.77
CT and TT	73	61	92	133	36	63	1.38	(0.97-1.95)	1.71	(1.13-2.58)	0.98	(0.61-1.57)	
**HO-1 A-413T (rs2071746)**													
AA	58	55	77	148	27	64	1.00	-	1.56	(0.97-2.51)	0.74	(0.43-1.27)	0.49
At and TT	124	129	154	243	58	140	1.25	(0.88-1.78)	1.41	(0.96-2.08)	0.71	(0.46-1.08)	

[A]P for interaction

[B] Adjusted for age and gender.
